# Successful robot-assisted laparoscopic resection of pheochromocytoma in a patient with dilated cardiomyopathy: A case report on extremely high-risk anesthesia management

**DOI:** 10.1097/MD.0000000000035467

**Published:** 2023-10-13

**Authors:** Long Huang, Jiarui Wu, Baorong Lian, Daxue Zhang, Yujia Zhai, Liming Cao

**Affiliations:** a Department of Anesthesiology, The Third Affiliated Hospital of Shenzhen University, Shenzhen, China; b The First School of Clinical Medicine, Guangdong Medical University, Zhanjiang, China; c Shantou University Medical College, Shantou University, Shantou, China; d School of Nursing, Anhui Medical University, Hefei, China; e Department of Neurology, The First Affiliated Hospital of Shenzhen University, Shenzhen, China; f College of Pharmacy, Changsha Medical University, Changsha, China; g Department of Neurology, The Third People’s Hospital of Yiyang City, Yiyang, China.

**Keywords:** anesthesia management, case report, dilated cardiomyopathy, hemodynamic monitoring, pheochromocytoma, preoperative assessment, robot-assisted laparoscopic resection

## Abstract

**Rationale:**

Anesthetic management during resection of pheochromocytoma is a huge challenge, especially when accompanied by dilated cardiomyopathy (DCM). However, there is a lack of research evidence in this area.

**Patient concerns:**

A 36-year-old man was admitted with a left retroperitoneal space-occupying lesion, present for 2 years. The patient also had DCM for 2 years. Blood analysis on admission showed elevated levels of norepinephrine and the N-terminus of the brain natriuretic peptide precursor. Abdominal computed tomography revealed a circular shadow in the left adrenal area. Echocardiography showed a cardiac ejection fraction of 31% to 37%, markedly enlarged left atrium and left ventricle, extensive cardiac hypokinesia, and reduced left ventricular diastolic and systolic functions.

**Diagnoses:**

The preoperative diagnosis was left paraganglioma/pheochromocytoma with DCM.

**Interventions:**

Multidisciplinary consultation, blood pressure measurements, and volume expansion measurements were performed preoperatively. Invasive arterial blood pressure, central venous pressure, depth of anesthesia, cardiac function, left heart volume, and body temperature were monitored intraoperatively.

**Outcomes:**

The adrenal pheochromocytoma was successfully removed, and the patient recovered well.

**Lessons:**

The anesthetic management for adrenal pheochromocytoma resection in adult patients with DCM is extremely high-risk but is evidently not impossible. Adequate preoperative evaluation and preparation, optimization of the anesthesia induction plan, close intraoperative monitoring of cardiac function and hemodynamic changes, and robot-assisted laparoscopic technology are the key success factors. The challenges to anesthetic management may be partly prevented with invasive monitoring techniques and minimally invasive surgery. This case confirms the importance of individual management and multidisciplinary cooperation for a successful outcome.

## 1. Introduction

Dilated cardiomyopathy (DCM) is a clinical condition characterized by left ventricular or biventricular dilation and impaired contractions that cannot be explained by abnormal loading conditions or coronary artery disease.^[1]^ The prevalence of DCM in the Chinese population is 19/100,000 persons.^[2]^ DCM has a poor prognosis, with a 5-year mortality rate of >50%.^[3]^ The anesthetic management of patients with DCM is challenging for anesthesiologists because of poor left systolic function, ventricular enlargement, the risk of malignant arrhythmias, and sudden cardiac death.^[4]^ Pheochromocytomas are rare neuroendocrine tumors; the serious and potentially lethal cardiovascular complications of these tumors are due to the potent effects of continuously or intermittently released catecholamines,^[5]^ causing persistent or paroxysmal hypertension and cardiac failure.^[6]^ In patients with pheochromocytomas, movement, endotracheal intubation, pneumoperitoneum, and intraoperative tumor compression can induce the release of large amounts of catecholamines. Severe intraoperative fluctuation in blood pressure is the main reason the resection of pheochromocytomas is considered a high-risk procedure. The plummeting of catecholamine levels after tumor resection can lead to hard-to-correct hypotension, especially during anesthetic induction or surgical removal of pheochromocytomas.^[6]^ However, the prognosis is excellent if the pheochromocytoma is promptly removed. Furthermore, patients with pheochromocytomas may have serious complications, such as DCM, which is highly challenging and necessitates higher requirements for preoperative assessment and anesthesia management.

In this report, we present a rare case of DCM in an adult patient who underwent extremely high-risk pheochromocytoma resection and review the relevant literature to improve anesthesia management.

## 2. Methods

This case report was approved by the Medical Ethics Committee of the Third Affiliated Hospital of Shenzhen University (No. 2023-LHQRMYY-KYLL-019). Informed consent was obtained from the patient.

### 2.1. Case report

A 36-year-old man was admitted for a left retroperitoneal space-occupying lesion that was detected on computed tomography (CT) performed during a general health evaluation 2 years earlier. The lesion had a diameter of 15 mm, and there were no other associated symptoms such as palpitations, fatigue, central obesity, or purple skin striae. A CT examination at a local hospital 2 weeks prior to presentation showed an obviously enlarged lesion of 65 mm in diameter, and laboratory tests showed elevated levels of catecholamines and their metabolites. Based on this, pheochromocytoma was considered. The patient also had DCM for 2 years and recently experienced shortness of breath while climbing stairs. The patient had no other relevant medical history.

### 2.2. Diagnostic assessment

The results of the physical examination at admission were as follows: height, 172 cm; weight, 89 kg; pulse, 78 bpm; blood pressure, 112/78 mm Hg; clear consciousness; regular heart rhythm; no moist rales in the lungs; and no edema in the lower limbs. No significant abnormalities were found in any other medical tests.

Blood analysis revealed normal platelet counts, white and red blood cell counts, coagulation parameters, liver and kidney function, blood electrolyte levels, and myocardial enzyme levels. Levels of direct renin (>500 mIU/L; reference range, 4.4–46.1 mIU/L) in the blood, 24-h urinary vanillylmandelic acid (24.77 mg/24 hours; reference range, <13.6 mg/24 hours), glycosylated hemoglobin (6.6%, reference range, 4.0–6.0%), fasting blood glucose (6.2 mmol/L; reference range, 3.9–6.1 mmol/L), and N-terminal of brain natriuretic peptide precursor (NT-proBNP, 1149–1950 pg/mL; reference range, <450 pg/mL) were elevated.

**Figure 1.**
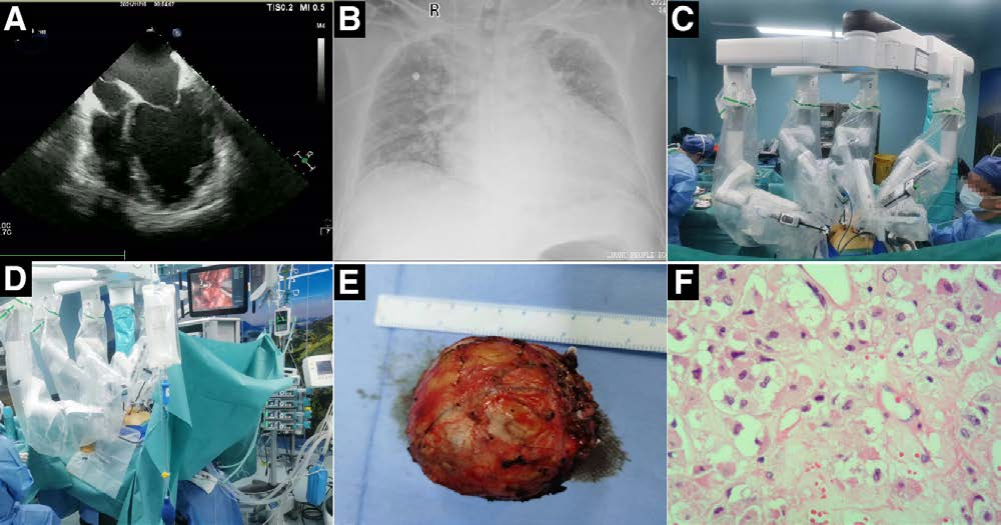
The main auxiliary examination results and surgical operation diagram of the patient. (A) Echocardiography showed a markedly enlarged left atrium and left ventricle. (B) Chest radiography revealed an enlarged cardiac shadow. (C and D) Da Vinci robot-assisted laparoscopic resection of the adrenal pheochromocytoma. (E) The resected ovoid mass (60 × 60 × 50 mm) with envelope visible by the naked eye. (F) Under a microscope (400 × magnification, hematoxylin-eosin staining), the pathology of the tumor showed cytoplasmic abundance presenting as granules or vacuoles, round or oval nuclei, and visible nucleoli, which are consistent with the features of a pheochromocytoma.

Abdominal CT showed an approximately 5.6 × 6.3 cm circular shadow in the left adrenal area, which was considered to be a pheochromocytoma or paraganglioma. Electrocardiography revealed a sinus rhythm and left anterior branch block. Echocardiography showed a cardiac ejection fraction of 31% to 37% (reference range: 55%–60%), left ventricular end-diastolic diameter of 71 mm (reference range: 45–55 mm), markedly enlarged left atrium and left ventricle (Fig. 1A), extensive cardiac hypokinesia, and reduced left ventricular diastolic and systolic function, consistent with the ultrasonographic changes of DCM. Chest radiography revealed an enlarged cardiac shadow (Fig. 1B).

The preoperative diagnosis was a left paraganglioma/pheochromocytoma with DCM. The patient underwent a Da Vinci robot (Intuitive Surgical, Sunnyvale, CA)-assisted (Fig. 1C and D) laparoscopic resection of the left adrenal pheochromocytoma. We intended to perform general anesthesia induction during endotracheal intubation, invasive arterial pressure monitoring, central venous pressure monitoring, intraoperative monitoring with transesophageal echocardiography (TEE), and bispectral index monitoring. Preoperative multidisciplinary consultation and preparation (phenoxybenzamine 20 mg/day, metoprolol 25 mg/day for 1 week, intravenous equilibrium liquid 500 mL/day, and hydroxyethyl starch 500 mL/day for 1 week. Echocardiography and NT-proBNP assessment were performed every 2 days; the blood pressure was lowered, and volume expansion therapy was administered for 1 week under continuous monitoring of the patient heart function. We observed the patient shortness of breath, monitored blood NT-proBNP levels, and evaluated inspiratory collapse of the inferior vena cava using ultrasound.

Anesthesia was induced by the intravenous administration of midazolam (1 mg), etomidate (20 mg), cis-atracurium (16 mg), dexmedetomidine (using a micropump, 0.5 µg/kg/h and for 30 minutes), and sufentanil (25 µg). Anesthesia was maintained using propofol (3–5 mg/kg/h), remifentanil (0.12 µg/kg/min), cis-atracurium (6 mg/h), and sevoflurane (1%) inhalation.

**Figure 2.**
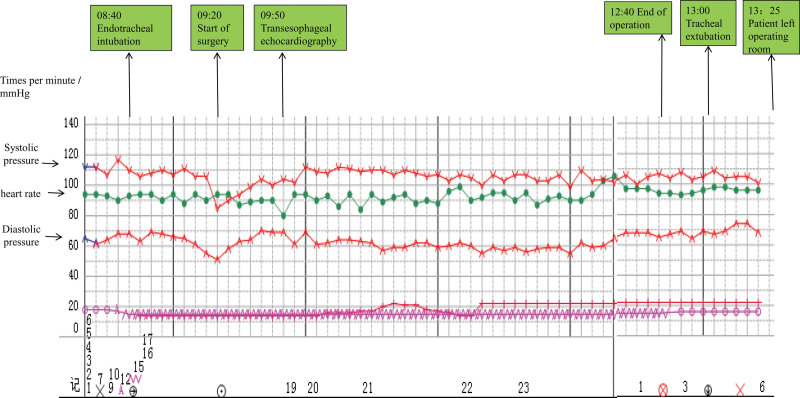
Milestones in anesthesia management and perioperative changes in blood pressure and heart rate.

The specific process of anesthesia induction comprised whole-course monitoring of the intraoperative cardiac function and volume state of the left heart using TEE, following general anesthesia with endotracheal intubation. Invasive arterial blood pressure, central venous pressure, depth of anesthesia, and temperature were monitored intraoperatively. Intraoperatively, we administered intravenous omeprazole to prevent stress ulcers and possible esophageal damage caused by the TEE probe and methylprednisolone to improve the stress response ability during the operation; we also administered human serum albumin. Intraoperative TEE showed a left ventricular ejection fraction of 28% to 32% and a normal inferior vena cava internal diameter (15–17 mm). The operative time was 3 hours 15 minutes, and the operative procedures were performed uneventfully. Intraoperative anesthesia was stable (Fig. 2), with the following parameters: invasive arterial blood pressure, 100 to 120 mm Hg/60 to 73 mm Hg; central venous pressure, 6 to 9 cmH_2_O; intraoperative blood loss, 150 mL; urine volume, 400 mL; and infusion volume, 1400 mL. The adrenal mass was successfully removed (Fig. 1E), and postoperative histopathology (Fig. 1F) of the tumor was consistent with pheochromocytoma. Postoperative analgesia (micro-pump butorphanol 0.1–0.3 mg/h for 2 days) was immediately performed under close monitoring. The patient was transferred to the intensive care unit for closer postoperative observation and transferred back to the general ward 3 days after the operation. He was discharged 4 days later with a good recovery. The patient was satisfied with his treatment and recovery.

## 3. Discussion

This report described the successful completion of a rare, high-risk operation with robotic assistance and the optimization of anesthesia management via careful preoperative evaluation and preparation, as well as intraoperative monitoring of cardiac function and hemodynamics to accurately guide fluid therapy and vasoactive drug administration. An efficient multidisciplinary team (hospital physicians, anesthetists, urologists, and cardiovascular specialists) plays a critical role in the management of patients with DCM and pheochromocytoma. The optimal anesthetic management of patients with DCM requires good preoperative assessment, close perioperative monitoring, suitable anesthesia, optimized fluid management, and a stable hemodynamic status.

### 3.1. Anesthesia preoperative assessment and preparation

Preoperative preparation for pheochromocytoma resection should meet the required high standards as much as possible. The specific requirements are as follows: blood pressure <120/80 mmHg; heart rate of 60 to 80 beat/min; electrocardiography showing ventricular premature contraction <1 beat/5 minutes and no ST-T changes; and vascular dilation and recovery of blood volume (hematocrit decreased by more than 5% after the volume expansion therapy); and improvement in the hypermetabolic syndrome and the correction of impaired glucose metabolism.^[7]^

High levels of catecholamines in the blood can cause the sustained contraction of blood vessels and reduce the effective circulating blood volume.^[8]^ Due to the long-term effects of catecholamines, patients with a pheochromocytoma often have high blood pressure, rapid heart rate, hypovolemia, and hemoconcentration. Adequate depressurization and volume expansion therapy should be performed preoperatively.^[9]^ However, our patient had DCM with poor left ventricular systolic function (the lowest ejection fraction was 31%); an infusion that is too rapidly administered or of too great a volume will increase cardiac preload and induce heart failure. After close monitoring of the patient cardiac function, the patient underwent smooth depressurization and prudent dilatation therapy for 1 week preoperatively. Guidelines recommend that all patients with hormonally functional paragangliomas undergo preoperative blockade with α-blockers as the first choice.^[9]^ The use of α receptor blockers^[10]^ combined with calcium antagonists may be more beneficial for stabilizing the intraoperative blood pressure.^[11]^ Preoperative preparation for adequate anesthesia is a prerequisite for stable circulation during pheochromocytoma resection.

### 3.2. Intraoperative management

The perioperative mortality of pheochromocytomas is high, and there is a risk of hypertensive crisis and heart failure.^[12]^ However, hypotension and hypoglycemic shock may develop after tumor resection. Moreover, the patient developed DCM. We optimized anesthesia induction and management by monitoring the depth of anesthesia, cardiac function, and hemodynamics (maintaining systolic and diastolic blood pressures at 90 to 140 mm Hg and 60 to 90 mm Hg, respectively). The patient was prone to hypotension and a rapid heart rate after anesthesia induction, and we administered micropump norepinephrine infusion while accelerating rehydration and maintaining blood glucose levels (at 6.0–11.0 mmol/L). The above modalities ensured that the surgery could be performed safely and smoothly.

### 3.3. Anesthesia induction and management

Anesthesia induction should be smooth with adequate sedation, analgesia, and muscle relaxation. We administered intravenous anesthesia and monitored the depth of anesthesia to ensure that it was stable and appropriate.

### 3.4. Monitoring of cardiac function, hemodynamics, and blood glucose levels

Perioperative hemodynamic instability remains the greatest surgical and anesthetic challenge in pheochromocytoma.^[13]^ As the patient in the present study had poor cardiac function, a TEE probe was inserted intraoperatively to monitor and evaluate left heart function and volume in real-time to guide intraoperative rehydration treatment. TEE is a useful tool that can continuously monitor the cardiac preload and cardiac hemodynamic parameters. Measurement of left ventricular size and ejection fraction remains central to risk stratification and treatment of DCM.^[14]^ Intraoperative TEE monitoring showed that the left ventricular ejection fraction was stable at 28% to 32% and that the inferior vena cava diameter was within the normal range.

Pheochromocytoma resection may be accompanied by significant hemodynamic fluctuations. Systolic pressure variations can guide fluid therapy during surgery,^[8]^ and careful blood pressure control can help prevent complications.^[15]^ There was no obvious increase in blood pressure and heart rate during tumor resection, which may be related to adequate preoperative preparation, decreased cardiac function, and the use of robot-assisted laparoscopy. The management of hemodynamics was guided by continuous monitoring of invasive arterial pressure and central venous pressure. Invasive arterial blood pressure monitoring allows real-time monitoring of blood pressure changes to quickly guide the application of vasoactive drugs during surgery. Arterial catheterization is also convenient for drawing blood for blood sugar and blood gas analysis during surgery, providing timely adjustment to maintain the acid-base balance and stabilizing serum electrolyte and blood sugar levels. Monitoring invasive arterial pressure and central venous pressure can help the assessment of hemodynamic changes and volume status, respectively, in real-time. However, this may increase the risks of vascular damage and infection, respectively.

Hypoglycemia can occur after the resection of pheochromocytoma and is more prevalent in patients with epinephrine-predominant neoplasms and longer operative times.^[16]^ We monitored blood glucose continuously to prevent hypoglycemia. Blood glucose levels should be closely monitored at least once every 6 hours after surgery, and hydrocortisone or methylprednisolone should be administered if necessary.

### 3.5. Fluid management strategies

Goal-directed fluid therapy was intraoperatively administered during surgery, and the intravascular volume was appropriately replenished. Fluid overload can lead to heart failure and pneumonia, and fluid restriction can lead to a drop in blood pressure or shock. Effective fluid management requires observation of central venous pressure, hemodynamics, urine volume, serum lactic acid, and pulmonary artery wedge pressure. Fluid resuscitation must be performed when postoperative hypotension occurs, and short-acting vasopressin is required if the blood volume is engorged.

### 3.6. Relationship between pheochromocytoma and DCM

Pheochromocytoma releases catecholamines, which may result in catecholamine-induced cardiomyopathy, including DCM,^[17]^ although this complication is rarely reported. The chronic overuse of an adrenaline inhaler may cause DCM.^[18]^ The catecholamine-induced vasoconstriction of small arterioles, a direct toxic effect of catecholamine and its metabolites, and direct receptor-mediated mechanisms are thought to contribute to cardiomyopathy in patients with pheochromocytoma.^[17,19]^ Pheochromocytoma may lead to myocardial fibrosis with both systolic and diastolic dysfunction.^[20]^ The impaired myocardial function can be improved by active medical treatment or surgery^[21]^ but may not be completely restored in some patients.

### 3.7. Operative treatment

The key management for catecholamine-induced cardiomyopathy associated with pheochromocytoma is early surgery. Laparoscopic tumor removal is the preferred procedure.^[5]^ Robot-assisted laparoscopy can effectively improve the perioperative condition of patients, reduce stress and energy metabolism, and reduce complications.^[22]^ Robot-assisted precision surgery can reduce the stimulation of pheochromocytomas, thereby reducing surgical complications and improving safety. Therefore, we proposed and successfully completed a Da Vinci robot-assisted laparoscopic resection of adrenal pheochromocytoma. There are reports of the reversibility of myocardial dysfunction with surgical treatment.^[21]^

### 3.8. Limitations

As this was a case report, the generalizability of our results is limited. Further research with a larger sample size is required to confirm our findings.

## 4. Conclusion

The anesthetic management for adrenal pheochromocytoma resection in adult patients with DCM is extremely high-risk but is evidently not impossible. Our multidisciplinary team conducted an adequate preoperative assessment and close perioperative monitoring; anesthesia and fluid management were optimized, and a stable hemodynamic state was achieved. Robot-assisted laparoscopy further improved surgical safety. The challenges to perioperative anesthetic management may be partly prevented with invasive monitoring techniques and minimally invasive surgery. This case confirms the importance of individual management and multidisciplinary cooperation for a successful outcome.

## Acknowledgments

We would like to thank the “Double-First Class” Application Characteristic Discipline of Hunan Province (Pharmaceutical Science) for its support.

## Author contributions

**Data curation:** Long Huang, Jiarui Wu.

**Formal analysis:** Long Huang, Jiarui Wu, Liming Cao.

**Funding acquisition:** Yujia Zhai, Liming Cao.

**Investigation:** Baorong Lian, Daxue Zhang.

**Visualization:** Long Huang, Jiarui Wu, Baorong Lian, Daxue Zhang.

**Writing – review & editing:** Yujia Zhai, Liming Cao.

**Writing – original draft:** Liming Cao.
